# ^1^H-NMR metabolomics reveals the Glabrescione B exacerbation of glycolytic metabolism beside the cell growth inhibitory effect in glioma

**DOI:** 10.1186/s12964-019-0421-8

**Published:** 2019-08-28

**Authors:** Giuseppina D’Alessandro, Deborah Quaglio, Lucia Monaco, Clotilde Lauro, Francesca Ghirga, Cinzia Ingallina, Michela De Martino, Sergio Fucile, Alessandra Porzia, Maria Amalia Di Castro, Federica Bellato, Francesca Mastrotto, Mattia Mori, Paola Infante, Paola Turano, Stefano Salmaso, Paolo Caliceti, Lucia Di Marcotullio, Bruno Botta, Veronica Ghini, Cristina Limatola

**Affiliations:** 1 Department of Physiology and Pharmacology, Sapienza University, Rome, Italy; 2 IRCCS Neuromed, Pozzilli, IS Italy; 3 Department of Chemistry and Technology of Drugs, “Department of Excellence 2018−2022”, Sapienza University of Rome, P.le Aldo Moro 5, 00185 Rome, Italy; 4 Center For Life Nano Science@Sapienza, Istituto Italiano di Tecnologia, Rome, Italy; 5 Department of Molecular Medicine, Laboratory affiliated to Istituto Pasteur Italia Fondazione Cenci Bolognetti, Sapienza University of Rome, Rome, Italy; 6 Department of Pharmaceutical and Pharmacological Sciences, University of Padova, Padova, Italy; 7 Department of Biotechnology, Chemistry and Pharmacy, “Department of Excellence 2018−2022”, University of Siena, via Aldo Moro 2, 53100 Siena, Italy; 8 CERM and Department of Chemistry, University of Florence, Via Luigi Sacconi 6, 50019 Sesto Fiorentino, Florence Italy; 9 CIRMMP, Via Luigi Sacconi 6, 50019 Sesto Fiorentino, Florence Italy; 10 Department of Physiology and Pharmacology, Laboratory affiliated to Istituto Pasteur Italia Fondazione Cenci Bolognetti, Sapienza University of Rome, Rome, Italy

**Keywords:** Glioma, Hh pathway, Isoflavones, ^1^H-NMR spectroscopy, Metabolomics

## Abstract

**Background:**

Glioma is the most common and primary brain tumors in adults. Despite the available multimodal therapies, glioma patients appear to have a poor prognosis. The Hedgehog (Hh) signaling is involved in tumorigenesis and emerged as a promising target for brain tumors. Glabrescione B (GlaB) has been recently identified as the first direct inhibitor of Gli1, the downstream effector of the pathway.

**Methods:**

We established the overexpression of Gli1 in murine glioma cells (GL261) and GlaB effect on cell viability. We used ^1^H-nuclear magnetic resonance (NMR) metabolomic approach to obtain informative metabolic snapshots of GL261 cells acquired at different time points during GlaB treatment. The activation of AMP activated protein Kinase (AMPK) induced by GlaB was established by western blot. After the orthotopic GL261 cells injection in the right striatum of C57BL6 mice and the intranasal (IN) GlaB/mPEG_5kDa_-Cholane treatment, the tumor growth was evaluated. The High Performance Liquid Chromatography (HPLC) combined with Mass Spectrometry (MS) was used to quantify GlaB in brain extracts of treated mice.

**Results:**

We found that GlaB affected the growth of murine glioma cells both in vitro and in vivo animal model. Using an untargeted ^1^H-NMR metabolomic approach, we found that GlaB stimulated the glycolytic metabolism in glioma, increasing lactate production. The high glycolytic rate could in part support the cytotoxic effects of GlaB, since the simultaneous blockade of lactate efflux with α-cyano-4-hydroxycinnamic acid (ACCA) affected glioma cell growth*.* According to the metabolomic data, we found that GlaB increased the phosphorylation of AMPK, a cellular energy sensor involved in the anabolic-to-catabolic transition.

**Conclusions:**

Our results indicate that GlaB inhibits glioma cell growth and exacerbates Warburg effect, increasing lactate production. In addition, the simultaneous blockade of Gli1 and lactate efflux amplifies the anti-tumor effect in vivo, providing new potential therapeutic strategy for this brain tumor.

**Graphical Abstract:**

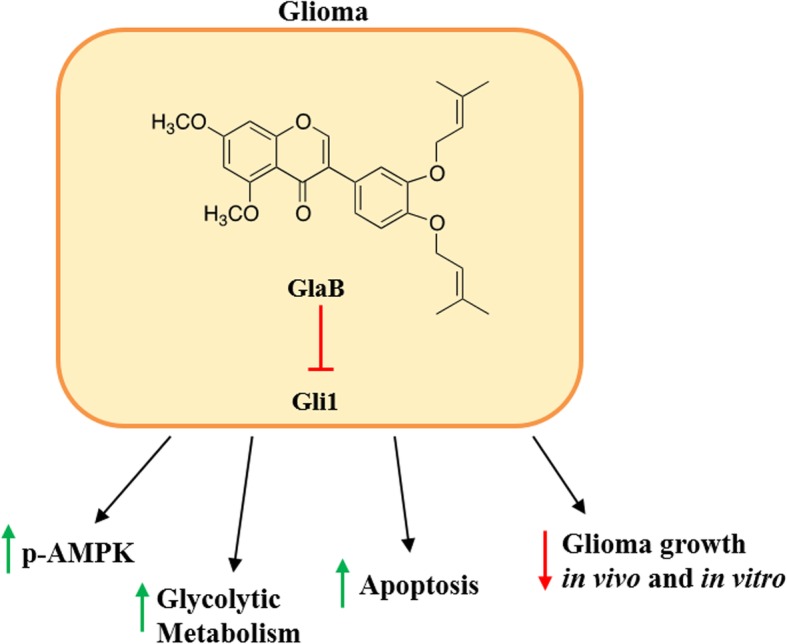

**Electronic supplementary material:**

The online version of this article (10.1186/s12964-019-0421-8) contains supplementary material, which is available to authorized users.

## Background

Glioblastoma (GBM) is the most frequent and lethal cancer originating in the central nervous system (CNS) in adults [1, 2]. Despite current treatments consisting of surgery followed by adjuvant radiotherapy and chemotherapy, GBM patients have a mean survival of 15 months from diagnosis [3]. Even if significant progresses in molecular targeting of brain tumors were made, several promising preclinical results revealed poor translatability, and there is urgent need of novel targeted therapies for patients. The Hedgehog (Hh) signaling pathway is involved in many physiological and pathological processes, including cancer, adipocyte differentiation, diabetes and obesity [4, 5]. In particular, the Hh signaling is implicated in tissue-patterning during embryonic development, in tissues repair and in epithelial-to-mesenchymal transition [6, 7]. The binding of Hh ligands (Sonic hedgehog (Shh), Indian hedgehog (Ihh), or Desert hedgehog (Dhh)) releases the inhibitory effect of Patched (Ptch), a transmembrane receptor, on Smoothened (Smo), which is also located on cell membrane [8]. The signaling cascade initiated by Smo leads to the activation and nuclear localization of the transcription factors of the zinc finger family Gli (Gli1, Gli2 and Gli3), which drive the expression of Hh target genes, mostly involved in proliferation, survival, and angiogenesis [9]. Alterations of the Hh-pathway contribute to tumorigenesis and tumor growth of several human cancers, including medulloblastoma [10], rhabdomyosarcoma [11], melanoma [12], basal cell carcinoma [13], and glioma [14], through several mechanisms. Although the role of Hh in the infiltrative growth of glioma remains to be elucidated, several studies claim its involvement in the promotion of cell proliferation, cancer stem cell self-renewal and tumor progression [15–17]. Thus, this pathway already emerged as a therapeutic target in oncology [18–20] and we have recently identified the first natural small molecule able to interfere with Gli activity [19], Glabrescione B (GlaB). In particular, GlaB binds to Gli1, and impairs its activity interfering with DNA interaction [19].

It is known that several malignant tumors, including GBM, consume high amounts of glucose at fast rate, with the production of lactic acid, even in the presence of oxygen (“Warburg effect”) [21–23]. To promote the high exploitation of glucose for rapid energy production through glycolytic flux and to avoid intracellular acidosis, cancer cells rapidly wipe out the lactic acid to the extracellular environment by the plasma membrane mono-carboxylate transporters (MCTs) [24]. Considering that lactate is a key metabolic driver implicated in the regulation of metabolic pathways, immune responses and cell-to-cell communication within the tumor microenvironment, targeting lactate metabolism and transport can represent a promising approach to counteract cancer growth and progression [25–27]. In this study, we found that GlaB treatment of GL261 glioma cells inhibits *Gli1* transcription, reducing glioma cell growth. Using an untargeted ^1^H-NMR metabolomic approach [28, 29], we also demonstrated that GlaB treatment increases both intra- and extracellular levels of lactate in GL261 cells, promoting the glycolytic process over the tricarboxylic acid cycle (TCA). Consistently, GlaB treatment induces the phosphorylation of a key protein involved in anabolic-catabolic transition, namely AMPK. The simultaneous blockade of lactate efflux with ACCA, a specific MCT inhibitor, further reduced glioma cell growth. These results were confirmed by an in vivo mouse model of glioma, thereby opening new perspectives for combination therapy in the treatment of this lethal tumor.

## Methods

### Materials

Cell culture medium (Dulbecco’s modified minimum essential medium, DMEM), fetal bovine serum (FBS), penicillin G, streptomycin, glutamine, sodium pyruvate and Hoechst were from GIBCO Invitrogen (Carlsbad, CA); rabbit anti p-AMPKα, AMPKα, were from Cell Signaling (Danvers, MA); anti mouse Gli1 was from Santa Cruz; 3-(4,5-Dimethylthiazol-2-yl)-2,5-diphenyltetrazolium bromide (MTT) salt, DMSO, Hematoxylin & Eosin were from Sigma-Aldrich (Saint Louis, MO). Glabrescione B was synthesized in our laboratory as previously described [30].

### Orthotopic tumor cell injection and intranasal treatment

Eight-week-old male mice were deeply anesthetized and placed in a stereotaxic head frame. Mice were injected with 1 × 10^5^ GL261 cells at 2 mm lateral and 1 mm anterior to the bregma in the right striatum. Cell suspensions, in sterile phosphate buffered saline (PBS) (4 μl) were injected with a Hamilton syringe at a rate of 1 μl/min at 3 mm depth. After 7 days, mice were intra-nasally treated with GlaB/mPEG_5kDa_-Cholane (1.44 mg/Kg, 40 μl), ACCA (33 mm, 6 μl) or mPEG_5kDa_-Cholane (40 μl) using the snorting delivery method. Briefly, mice anaesthetized and maintained with 1.5% isofluorane (Esteve, UK) were laid on their back. Suspensions were administered to mice, 3 μl drop at a time, alternating the nostrils, with a lapse of 1 min between each administration. GlaB/mPEG_5kDa_-Cholane treatment was repeated six times at 2-day intervals. ACCA treatment was daily.

### Tumor volume evaluation

Brains were isolated and fixed in 4% buffered p-formaldehyde 22 days after GL261 injection. Coronal brain sections (20 μm) were prepared by standard procedures and collected every 100 μm. Slices were stained with hematoxylin and eosin as detailed by the manufacturer and tumor area were calculated by the Image Tool 3.0 software (University of Texas, Health Science Center, San Antonio, TX, USA). Tumor volume was calculated according to the formula (volume = t × ΣA), where A = tumor area/slice and t = thickness.

### Cell culture

GL261 cells were kindly provided by Dr. Serena Pellegatta, Neurological Institute “Carlo Besta”, Italy. GL261 were cultured in DMEM supplemented with 20% heat-inactivated FBS, 100 IU/ml penicillin G, 100 μg/ml streptomycin, 2.5 μg/ml amphotericin B, 2 mm glutamine under the form of L-alanyl-L-glutamine, and 1 mm sodium pyruvate, at 37 °C in a 5% CO_2_ humidified atmosphere.

### MTT assay

GL261 cells were plated in 96 well plates (5000/well) in 100 μl DMEM + 1% FBS and incubated in the absence or presence of GlaB (5 μm). After 24 h, 48 h, 72 h and 96 h, 10 μl MTT (5 mg/ml) were added to culture medium and the plate incubated at 37 °C for 90 min. After incubation, the medium was removed and the cells were solubilized with 100 μl DMSO. Formazan produced by viable cells was read on microplate reader (Bio-Tek Instruments, USA) at absorbance of 562–530 nm.

### Immunofluorescence

GL261 cells (1 × 10^5^/ well) or pure primary astrocytes were plated in 24 well plates on glass coverslip. After 48 h, cells were fixed with paraformaldehyde, permeabilized with 0.2% Triton-X-100, blocked with 1% BSA-PBS and incubated O/N at 4 °C with a mouse monoclonal antibody against mouse Gli1 in 0.1% BSA-PBS (1:200, sc-515,751, Santa Cruz Biotechnology, CA, USA). The specific protein was visualized using a secondary antibody coupled to a fluorescent marker (1:2000 Alexa anti mouse#594 in 0.1%BS-PBS, 1 h at RT). Nuclei were stained with Hoechst 33258 (Molecular Probes, Life Technologies, USA) and examined by fluorescence microscopy. The images were digitized using a CoolSNAP camera (Photometrics) coupled to an ECLIPSE Ti-S microscope (Nikon) and processed using MetaMorph 7.6.5.0 image analysis software (Molecular Device). Immunofluorescence intensity was quantified by the integrated intensity density method on automatic threshold analysis.

### RNA preparation and qRT-PCR analysis

Total RNA was isolated from cell cultures using Trizol reagent (Ambion, Life Technologies, USA) according to the manufacturer’s instructions. The cDNA was prepared using the iScript Reverse Transcription Supermix (Bio-Rad Laboratories, USA); the quantitative PCR was performed using the SsoFast Evagreen Supermix (Bio-Rad Laboratories, USA) according to the protocol for use in the Biorad I cycler System. For the quantification analysis, the comparative threshold cycle (Ct) method was used. The Ct values of each gene were normalized to the Ct value of *Gapdh* in the same RNA sample. The gene expression levels were evaluated by fold change using the eq. 2^-ddCt^. Primers used: *Gli1* forward: TGAAAACCTCAAGACGCACC; *Gli1* reverse: ACGTATGGCTTCTCATTGGAG; *Gapdh* forward: TCGTCCCGTAGACAAAATGG; *Gapdh* reverse: TTGAGGTCAATGAAGGGGTC.

### Western blot

For protein analysis, GL261 cells (2 × 10^5^) were seeded on 12 well plates and treated with GlaB 5 μm for different times (5–10–20-30 min), washed with PBS and lysed in hot 2x Laemmli buffer, boiled 5 min and sonicated. The same amount of proteins was separated on 10% SDS-polyacrylamide gel electrophoresis and analyzed by western immunoblot using the following primary antibodies: P-AMPKα (1:2500, Cell Signaling), AMPKα (1:2000, Cell Signaling); HRP-tagged goat anti-mouse and anti-rabbit IgG were used as a secondary antibody (1:2000; Dako). Detection was performed through the chemiluminescent assay Immun-Star Western C Kit (Bio-Rad, CA) and densitometric analysis was carried out with Quantity One software (Bio-Rad, CA).

### FACS

The FITC-Annexin V Apoptosis Detection Kit (BD Bioscience, Germany) was used according to the manufacturer’s protocol. In brief, treated GL261 cells were detached and washed twice with cold PBS and resuspended in binding buffer at a final density of 10^6^ cells/ml. FITC-Annexin (5 μl) and PI (5 μl) were added to 100 μl of the cell suspension containing 10^5^ cells. The cell suspension was mixed by gentle vortexing and then incubated for 15 min at room temperature in the dark. Then binding buffer were added and cells were analyzed by flow cytometry using FACS Calibur (BD Bioscience, Germany) and the FloWJo software (BD Bioscience).

### GL261 cell culture for NMR-metabolomic analysis

For the metabolomic analysis, five independent experiments were performed. In each experiment, confluent GL261 cells were trypsinized, counted and plated (3 × 10^6^) in 6 cm dishes containing 1% FBS medium. After 4 h the growth medium was changed and supplemented with GlaB 5 μm or vehicle (DMSO) for 12 h or 24 h or 48 h. In all cases, the culture medium of each dish was collected, centrifuged (10,000 g for 10 min) and immediately stored at − 80 °C for the *exo*-metabolome analysis. On the other side, cells were extensively washed (4 times) with ice-cold phosphate-buffered saline (PBS 1X), in order to completely remove any residue of culture medium, trypsinized and centrifuged. Afterwards, cells were dispersed in a buffer solution containing 10 mm Tris (pH 7.4), 5 mm EDTA, 120 mm NaCl, and protease inhibitors (Complete Protease inhibitor Cocktail tablet, Roche). Cell lysis was obtained by sonication and the cytosolic fraction containing the metabolites was obtained upon centrifugation at 17,000 g for 1 h at 4 °C. Subsequently, cytosols were collected for analysis and stored at − 80 °C for the *endo*-metabolome analysis.

### NMR sample preparation

Frozen samples were thawed at room temperature and shaken before use. NMR cell lysate samples were prepared into 5.00 mm NMR tubes (Bruker BioSpin srl) after the addition of 50 μl of ^2^H_2_O containing 10 mm sodium 3-trimethylsilyl[2,2,3,3-^2^H_4_] propionate (TMSP) to 450 μl of cell lysate. In the case of cell culture media, an aliquot of 300 μl of a sodium phosphate buffer (70 mm Na_2_HPO_4_; 20% v/v ^2^H_2_O; 4.6 mm TMSP, pH was adjusted to the final value of 7.4 using 1 m HCl) were added to 300 μl of each medium sample and the mixture was homogenized by vortexing for 30 s (s). The mixture was transferred into a 5.00 mm NMR tubes (Bruker BioSpin srl) for analysis.

### NMR experiments

In order to study the possible metabolomic changes induced by GlaB treatment, ^1^H-NMR spectra were acquired on both cell lysates and conditioned media verifying their feasibility and reproducibility (Additional file 1: Figure S1). NMR spectra were recorded with a Bruker 900-MHz spectrometer equipped with CP TCI ^1^H/^13^C/^15^N probe. The ^1^H-NMR spectra of cell lysates were acquired with the Carr-Purcell-Meiboom-Gill (CPMG) sequence using a monodimensional spin–echo sequence with water presaturation (cpmgpr, Bruker). 256 scans over a spectral region of 18 kHz were collected into 110 K points, giving an acquisition time of 3.07 s. The spectra were recorded with the CPMG pulse sequence [31] to impose a T_2_ filter that allows selective observation of small molecular weight components. The total T_2_ delay was set to 290 ms. The T_2_ filtering in the CPMG pulse sequence, which contains trains of -(τ-180°-τ)- blocks repeated *n* = 256 times, was achieved with a total spin-echo delay (2nτ) of 80 ms. The acquisition of each spectrum required about 32 min. The ^1^H-NMR spectra of conditioned media were acquired with a 1D nuclear Overhauser enhancement spectroscopy (NOESY)-presaturation pulse sequence (noesygppr1d, Bruker). A total of 64 scans with 110 K data points were collected using a spectral width of 17,942 Hz, an acquisition time of 3.07 s, a relaxation delay of 4 s, and a mixing time of 100 ms.

The raw data were multiplied by a 0.3 Hz exponential line broadening before applying Fourier transform. Transformed spectra were automatically corrected for phase and baseline distortions and calibrated (chemical shift was referenced to the proton of TMSP at δ 0.0 ppm.) using TopSpin 3.5 (Bruker Biospin srl).

#### Spectral analysis

Each spectrum in the region 10.00–0.2 ppm was segmented into 0.02 ppm chemical shift bins, and the corresponding spectral areas were integrated using the AMIX software (Bruker). The area of each bin was normalized to the total spectral area [32], calculated with the exclusion, for the cell lysates, of the regions 2.55–2.57, 2.69–2.72, 3.20–3.24, 3.6–3.65, 3.67–3.72, 3.75–3.78, 3.79–3.82, 3.86–3.9, 7.65–7.70, 8.05–8.13 ppm (which correspond to EDTA peaks, Tris peak, and the most intense peaks of the protease inhibitors used for sample preparation) and 4.5–5.5 ppm (water region). For the conditioned media exclusion regarded only the water region (4.5–5.0 ppm).

#### Metabolite statistical analysis

The multivariate and univariate statistical analyses were performed using R 3.0.2 in house scripts. Multivariate statistical analysis was performed on the obtained buckets; unsupervised principal component analysis (PCA) was used to obtain an overview of the data (visualization in a reduced space, clusters detection, screening for out-layers). The global accuracy for classification was assessed by means of a leave-one-out cross-validation scheme (LOOCV).

Twenty-nine and twenty-seven metabolites for cell lysates and conditioned media, respectively, were assigned (Additional file 2: Table S1) and their levels analyzed. The assignment procedure was made up using a NMR spectra library of pure organic compounds, public databases (e.g. Human Metabolome Database-HMBD, Additional file 2: Table S1), stored reference NMR spectra of metabolites, spiking NMR experiments and using data available in literature. Matching between new NMR data and databases was performed using the AMIX software. The relative concentrations of the various metabolites were calculated by integrating the corresponding signals in the spectra with an in-house script. The non-parametric Wilcoxon test was used for the determination of the meaningful metabolites: a *p*-value of 0.05 was considered statistically significant.

### GlaB brain level concentration measured by HPLC coupled with electrospray mass spectrometry

#### Sample preparation

HPLC-MS analyses were performed on three independent experiments of brain samples from mice treated with GlaB/mPEG_5kDa_-Cholane. The brain sample (500 mg), previously stored at − 80 °C for 24 h, was homogenized with 1 ml of a Phosphate Buffered Saline solution, PBS, using an ultrasound probe. A solution of ZnSO_4_ (0.1 m in H_2_O, 1 ml) and acetonitrile for HPLC (1 ml) was added to the mixture, which was further homogenized with ultrasound for 15 min. Subsequently, the homogenate brain was stirred for 1 h at room temperature and was then centrifuged at 3000 g for 5 min at T = 4 °C. The supernatant was collected. The following procedure to obtain the brain extract was repeated twice. Acetonitrile for HPLC (1 ml) was added to the brain residue, stirred for 30 min at room temperature, then centrifuged at 3000 g for 5 min at T = 4 °C and the supernatant collected. Brain extract samples were centrifuged for 5 min at 5000 g and 20 μl of supernatant injected into the HPLC-MS without further treatments.

#### Equipment and chromatographic conditions

The HPLC chromatographic system used was an UltiMate 3000 RS system (Thermo Fisher Dionex Sunnyvale, California), equipped with an UltiMate 3000 LPG-3400RS Low Pressure Mixing Biocompatible Gradient Pump, an UltiMate 3000 WPS-3000TSL Analytical Autosampler with a thermostated analytical scale in-line split loop injection principle, a thermostated column ventilated compartment (temperature range: 5–110 °C) and a diode array detector (UltiMate 3000 DAD-3000RS Rapid Separation Diode Array Detector, up to 200 Hz acquisition rate) with a low dispersion 13 μl flow cell. The stationary phase used was a Titan C18 (100 × 3.0 mm LxI.D. 1.9 μm). All chromatographic runs were performed at a flow-rate of 0.4 ml/min with the column equilibrated at 35 °C. Solvent A was Water/Acetonitrile 90:10 with 0.1% v/v of formic acid, and solvent B was Acetonitrile/Methanol 50:50 with 0.1% v/v of formic acid. The gradient was set as shown in Additional file 2: Table S2. The LC was directly interfaced to electrospray ionization (ESI) source coupled with a Single Quadrupole-MSQ Plus Detector. Ion source was operated in positive ESI mode and both Full Scan and SIM (m/z = 451, corresponding to the most abundant ion [M + H]^+^ of GlaB, from 22 to 27 min) were acquired for each sample. Optimal instrument source parameters for ionization were a cone voltage of 100 V and a Probe Temperature of 550 °C.

#### Sample quantification

Calibration curves were built both in UV (254 nm) and MS in SIM mode, by monitoring the m/z = 451 corresponding to the most abundant ion [M + H]^+^ of GlaB.

Calibration standards ranged from 0.743 to 7.938 μg/ml for UV curve (y = 0.2379x-0.0779, R^2^ = 1) with a Limit of Quantitation, LOQ, (defined for a signal-to-noise ratio > 10) of 1.48 ng on column, while for MS plot the lower range is 0.309 μg/ml showing a good linearity (y = 11,655x-2890.2, R^2^ = 1) with a LOQ of 0.62 ng on column (Additional file 1: Figure S2).

A known amount (0.45 μg/ml) of the GlaB analyte (a spike) was added to treated brain sample. The samples and the samples plus spike were then analyzed (Additional file 1: Figure S3). The sample with the spike will show a larger analytical response than the original sample due to the additional amount of analyte added to it. The difference in analytical response between the spiked and unspiked samples is due to the amount of analyte in the spike. This provides a calibration point to determine the analyte concentration in the original sample.

## Results

### GL261 cells overexpress *Gli1* and its inhibition with GlaB induces cell apoptosis

**Fig. 1:**
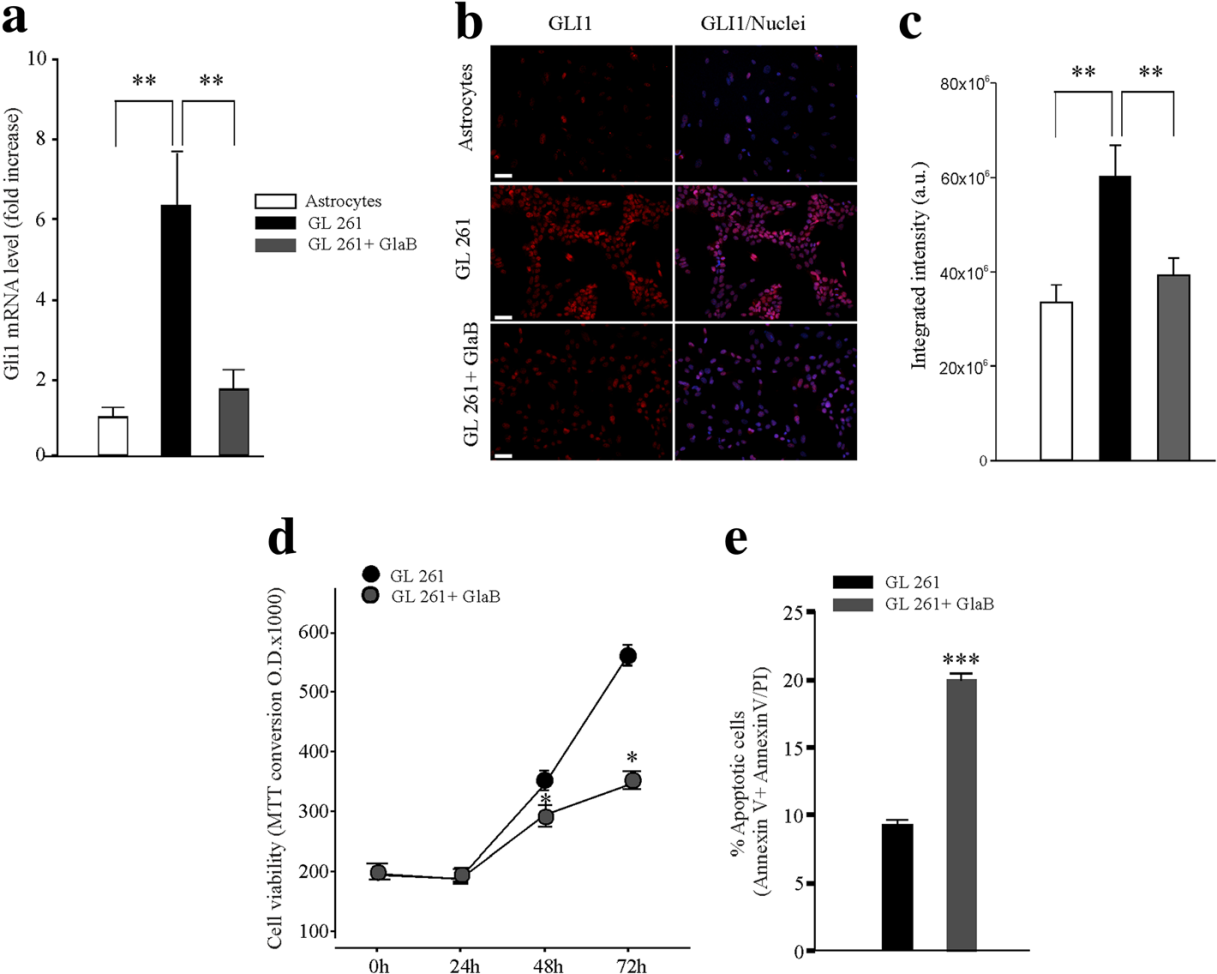
Murine glioma GL261 cells are Hedgehog dependent tumor cells. **a** Data (mean ± se) are normalized to *Gapdh* mRNA expression and are expressed as *Gli1* mRNA fold increase *vs* *Gli1* mRNA expression levels on astrocytes. One-way ANOVA, **p < 0.01, *n* = 7. **b** Immunofluorescence images of Gli1 protein (RED) in Astrocytes, GL261 and GL261 + GlaB treated (5 μm, 48 h) cells. **c** Protein signal quantification in Astrocytes, GL261 and GL261 + GlaB treated (5 μm, 48 h) cells. Nuclei were visualized by Hoechst (BLU) scale bar: 20 μm. One-way ANOVA, **p < 0.01, *n* = 10. **d** MTT growth curves in untreated and GlaB treated cells (5 μm, 24 h, 48 h, 72 h) data are expressed as mean ± se, *p < 0.05, *n* = 3 (each in quadruplicate). **e** Apoptosis measured as the sum of cells positive for Annexin V and Annexin V/PI double positive cells in GL261 untreated or treated with GlaB 5 μm for 48 h, data are expressed as mean ± se, ***p < 0.001, *n* = 3 (each in duplicate)

In order to investigate Hh signaling in glioma, we measured the expression of Gli1 by RT-PCR and by immunofluorescence. The mRNA expression of *Gli1* is shown in Fig. 1a, with GL261 cells expressing about 6-fold higher level of *Gli1* than primary astrocytes. GlaB treatment (5 μm, 48 h) significantly decreased *Gli1* transcription, indicating that the Hh signaling has a basal activity and that Gli1 is functionally expressed in these cells. Qualitatively similar results were obtained by immunofluorescence analyses (Fig. 1b and c). Staining with secondary antibody alone was performed as a control for specificity. In order to investigate the role of Gli1 in GL261 proliferation, we evaluated the effect of GlaB treatment (5 μm) on cell viability. As shown in Fig. 1d, GlaB treatment (48 h–72 h) significantly reduced cell viability. It has been shown that Gli1 inhibition could promote apoptosis in other cancer cells [33, 34]. We investigated whether GlaB treatment could induce programmed cell death in GL261 cells, by cytofluorimetric analysis of Annexin V staining and Propidium Iodide (PI) incorporation. Figure 1e shows that GlaB increased apoptosis at 48 h (measured as the sum of early -Annexin V positive- and late apoptotic -Annexin V + Annexin V/PI- positive cells) compared to control cells, indicating that GlaB treatment reduces cell proliferation and promotes apoptosis in glioma cells.

### GlaB treatment affects the metabolomic profiles of GL261 cells, exacerbating the Warburg effect

**Fig. 2:**
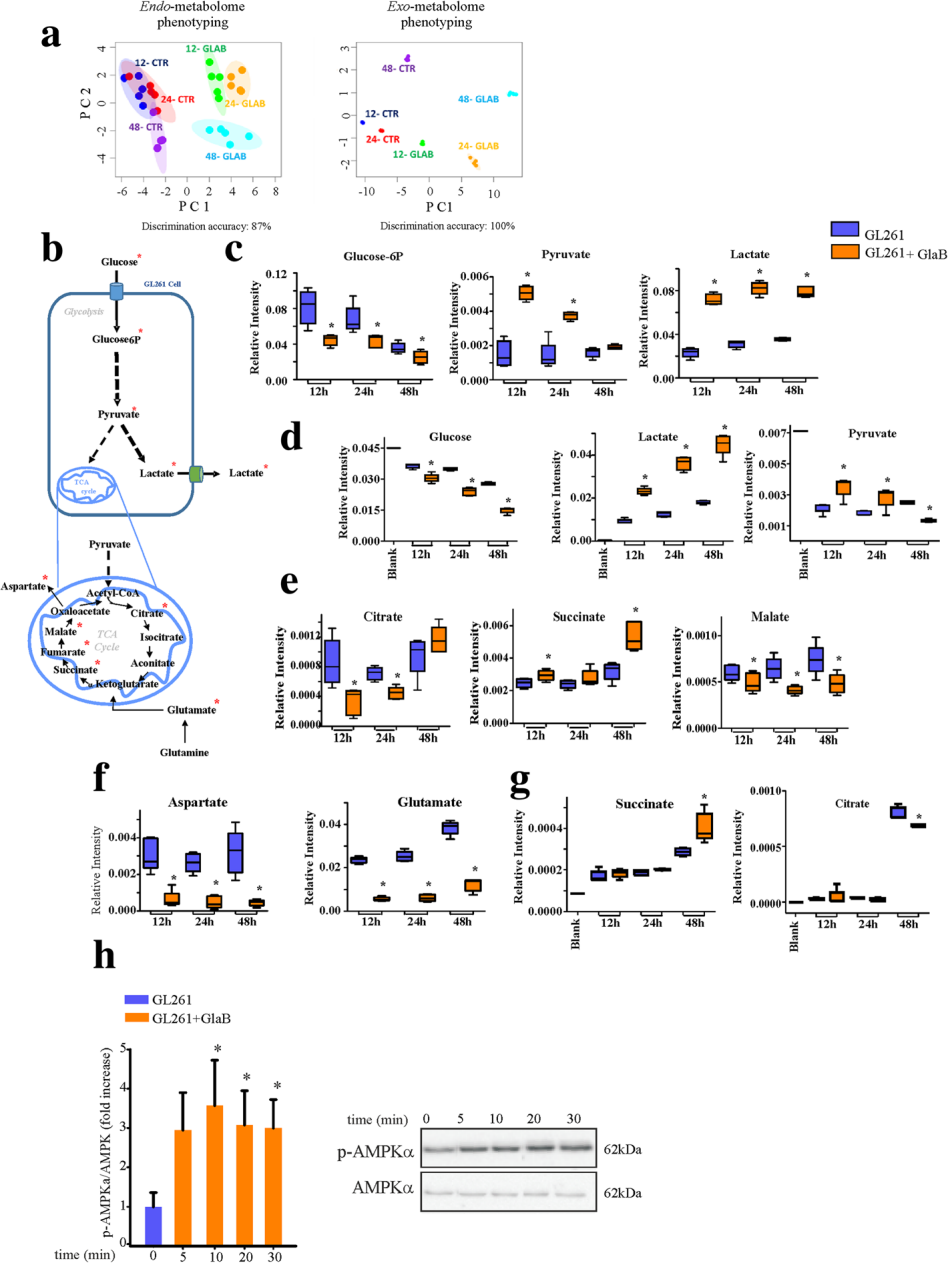
GlaB treatment significantly altered (*) the metabolic profile of GL261 cells. **a** Metabolomic phenotyping of cell lysates (left panel, *endo*-metabolome) and respective growth media (right panel, *exo*-metabolome) after 12 h, 24 h and 48 h of GlaB treatment. Score plots of PCA: PC1 *vs* PC2. The 6-group discrimination accuracy values are also reported. In the score plots, each dot represents a different sample, and each color represents a different group of samples: blue dots, 12-CTR; red dots, 24-CTR; purple dots, 48-CTR; green dots, 12-GLAB; orange dots, 24-GLAB; cyan dots, 48-GLAB. **b** Schematic representation of glycolysis and TCA cycle in GL261 cells. **c** Box plots showing the altered intracellular glycolytic metabolites over time. **d** Box plots showing the altered extracellular glycolytic metabolites over time. **e** Box plots showing the altered intracellular TCA-metabolites over time. **f** Box plots showing the altered intracellular TCA-related metabolites over time. **g** Box plots showing the altered extracellular TCA-metabolites over time. Data are expressed as mean ± se, *n* = 5 *p < 0.05 *vs* respective control. **h** Western blot analysis of GlaB treated (5 μm) GL261 cells at indicated time point for AMPK phosphorylation. Quantification of protein expression by densitometry analysis of the bands. Data are expressed as fold increase over control of phospho/total protein ratio of Ampk. Statistical analysis: One-way ANOVA followed by Dunn’s post hoc test *p ≤ 0.05. On the right, representative western blots

To investigate possible metabolic effects of GlaB in GL261 cells, we performed an untargeted ^1^H-NMR metabolic profiling on cellular lysates and growth media. ^1^H-NMR spectra were acquired in order to determine the metabolic fingerprints of GL261 untreated and GlaB-treated cells, at different time points (12 h, 24 h, and 48 h). Multivariate unsupervised PCA analysis performed on the acquired ^1^H-NMR spectra, clearly shows the strong remodeling of the metabolomic phenotyping of GL261 cells, upon GlaB treatment. Both the *endo-* and the *exo-*metabolome of GL261 cells were significantly modified by GlaB administration, with a discrimination accuracy among the six different groups ranging from 86.7% (lysates) to 100% (growing media) (Fig. 2a). The alteration of GL261 metabolomic profile was evident already after 12 h of treatment, both in the cell lysates and in the growth media. The 95% confidence level ellipses (Fig. 2a) for cell lysates at 12 h and 24 h are almost overlapping in untreated cells, while they are better separated, even if still close, in treated cells. The separation increases at 48 h, again with larger differences for the treated cell lysates, indicating that the metabolomic profile of cells was heavily altered by GlaB. An analogous trend is observed for media, although they are characterized by more homogeneous composition within each group (Fig. 2a). The observed trends suggest the presence of a sort of biphasic behavior, which reflects the effects on cell viability in Fig. 1c. When cell viability remains constant in untreated cells, the metabolome undergoes only minor shifts. The metabolic drift is more accentuated in GlaB-treated cells, where the initial effect of the drug becomes visible. At 48 h both cell types are in a growing phase, but GlaB-treated cells showed higher frequency of apoptotic cells compared to control (Fig. 1e). The Additional file 2: Table S1 reports all the assigned metabolites both in the cell lysates and in the extracellular media. The changes in metabolite concentrations for each time point are shown in Fig. 2 and in the Additional file 1: Figure S4 and S5, as they result from univariate analyses, and reflect the multiphase behavior described above. In GlaB-treated cells we found that the levels of several *endo*-metabolites are lower than in the untreated controls, both at 12 h and 24 h. These metabolites include glucose-6-phosphate, some amino acids (*e.g*, isoleucine, leucine, valine, phenylalanine, histidine, tyrosine, threonine, glutamate, aspartate, arginine, glutamine, N-acetylaspartate) and other molecules (e.g. choline, cadaverine, AXP-shorthand to indicate the not unambiguously assigned signals of ATP, ADP and AMP, formate, myo-inositol, creatine and creatinephosphate) (Fig. 2c, and Additional file 1: Figure S4). On the contrary, the levels of the endogenous products of the aerobic glycolysis (e.g. pyruvate, lactate and alanine) increased significantly (Fig. 2c, and Additional file 1: Figure S4). The behavior of the metabolites of the TCA cycle is more complex with decreased levels of citrate and malate, and increased levels of succinate and fumarate (Fig. 2e and Additional file 1: Figure S4).

The univariate statistical analysis of fresh and spent culture media also revealed essential differences in the uptake and release of metabolites: GlaB treatment for 12 h and 24 h significantly enhanced the release into the media of lactate and alanine. In contrast, the treatment enhanced the consumption of extracellular glucose over time. Pyruvate uptake was lower after 12 h and 24 h of treatment (Fig. 2d and Additional file 1: Figure S5). The uptake of the amino acids isoleucine and leucine decreased starting from 12 h of GlaB administration. Finally, the net changes in glycine levels go into opposite directions in treated and untreated cells; in the former, glycine is taken up as a consequence of GlaB treatment, while in the latter it is released into the media (Additional file 1: Figure S5). At later time point (48 h), stronger variations are observed: pyruvate uptake is larger in treated *vs* untreated cells, while its intracellular levels are similar. The extracellular levels of lactate in GlaB-treated cells increase significantly (Fig. 2c and d), as well as the intracellular alanine (Additional file 1: Figure S4). The extracellular levels of tyrosine, phenylalanine and histidine remain constant at 24 h and 48 h, while their intracellular levels are decreased in treated and untreated cells, suggesting their augmented consumption to sustain glioma growth. Intracellular leucine, isoleucine, valine also decrease in GlaB-treated cells, while a corresponding reduction of extracellular consumption is observed (Additional file 1: Figure S4 and S5).

Intracellular AXP levels drop upon GlaB treatment at 12 h and 24 h (Additional file 1: Figure S4). At 48 h, a significant increase of glutamine excretion is observed (Additional file 1: Figure S5).

As concern the TCA cycle metabolites, particularly relevant is the increase of succinate at the intra- and extracellular levels in GlaB treated samples, and the increase of citrate at extracellular level in both conditions (Fig. 2e and g).

Additionally, in cell lysates, significant decrements of the broad signals derived from macromolecule spectral components (e.g., lipids, lipoproteins and proteins) can be detected at 48 h of treatment (Additional file 1: Figure S6).

The anabolic-catabolic transition, together with the reduction of AXP levels, (Additional file 1: Figure S5) induced by GlaB treatment of GL261 cells, suggests that this molecule can favor energy stress in glioma. This hypothesis is further supported by the increased phosphorylation levels of the energy sensor AMPK. Indeed, data shown in Fig. 2h demonstrate that GlaB treatment (5 μm) significantly changed the phosphorylation level of this protein.

### GlaB/ACCA co-treatment induces GL261 cell necrosis

Since GlaB-treated GL261 cells show an increased Warburg-like metabolism, we investigated if the corresponding increase of lactate was involved in GlaB-induced modulation of cell viability. At this aim, cells were treated with ACCA (250 μm), a small-molecule competitive inhibitor of lactate transporters, and analyzed for cell growth, apoptosis and necrosis in the presence or absence of GlaB (5 μm). Previous studies demonstrated that ACCA induced tumor cell death, both in vivo and in vitro [25, 26].

**Fig. 3:**
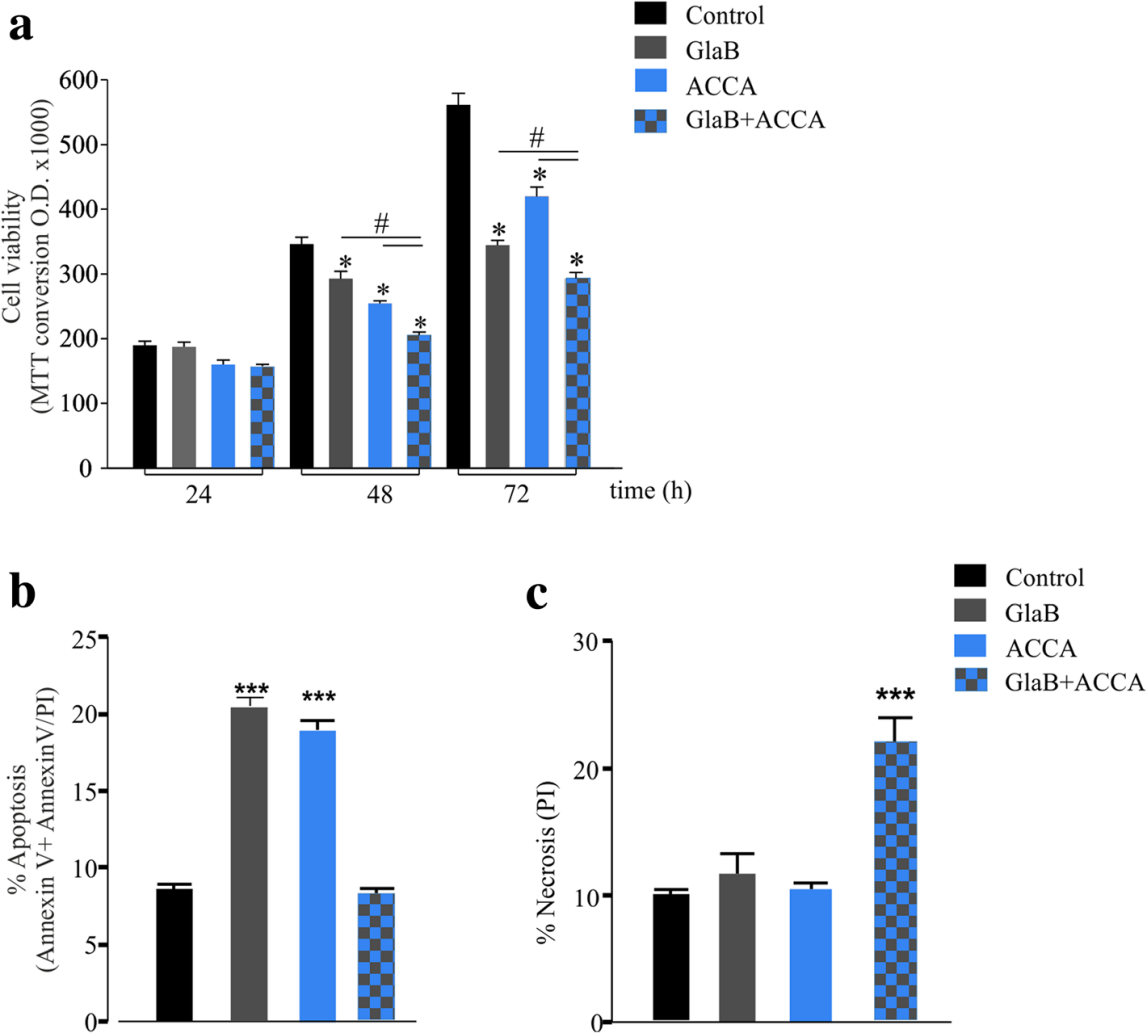
Blockade of lactate transport increases GlaB effect. **a** MTT assay showing that ACCA treatment (250 μm) and GlaB treatment (5 μm) at indicated time points reduced growth of GL261 cells and that this effect was higher when cells were treated with both. Data are expressed as mean ± se, *n* = 3 (each in quadruplicate), *p < 0.05 *vs* control, #*p* < 0.05 *vs* GlaB+ACCA by One-way ANOVA followed by Student-Neuman-Keuls (SNK) post-test. **b** Cytofluorimetry study showing that the treatment of GL261 with ACCA (100 μm, 48 h) increases the percentage of apoptotic cells (*n* = 4, One-way ANOVA, SNK test, data are expressed as mean ± se, ***p < 0.001 *vs* control). **c** Cytofluorimetry study showing that the co-treatment of GL261 with GlaB and ACCA (5 μm and 100 μm respectively) for 48 h increases the percentage of cells featuring non-apoptotic/necrosis like death, measured with Propidium Iodide (PI) staining (*n* = 4, One-way ANOVA, SNK test, ***p < 0.001 *vs* control)

As shown in Fig. 3a, ACCA treatment impairs GL261 viability, similarly to GlaB, and this effect was potentiated in GlaB/ACCA treated cells. ACCA treatment increased GL261 apoptosis (48 h), seen as Annexin V positive + Annexin V/PI positive cells (Fig. 3b and Additional file 1: Figure S7), as already reported for other glioma cells [25]. Co-treatment with GlaB/ACCA occluded the induction of apoptosis (Fig. 3b and Additional file 1: Figure S7) but switched the effect on cell viability to cell necrosis (PI positive cells) (Fig. 3c and Additional file 1: Figure S7). These data support the hypothesis that the increased glycolytic flux induced by GlaB promotes apoptosis in glioma cells, and that the blockade of lactate efflux transforms an otherwise programmed cell death in a less regulated, necrotic process.

### Effect of GlaB and GlaB/ACCA treatment in mouse model of glioma

To verify the translational potential of GlaB effects on tumor growth, we used a mouse model of glioma. C57BL6 mice were intracerebrally injected with syngeneic GL261 cells in the right striatum and, after 7 days, animals were intranasally treated with GlaB (1.44 mg/Kg, drug formulated with mPEG_5kDa_-Cholane, see methods), every two days, for six times, alone or in combination with ACCA (33 mm, 6 μl) administered every day.

**Fig. 4:**
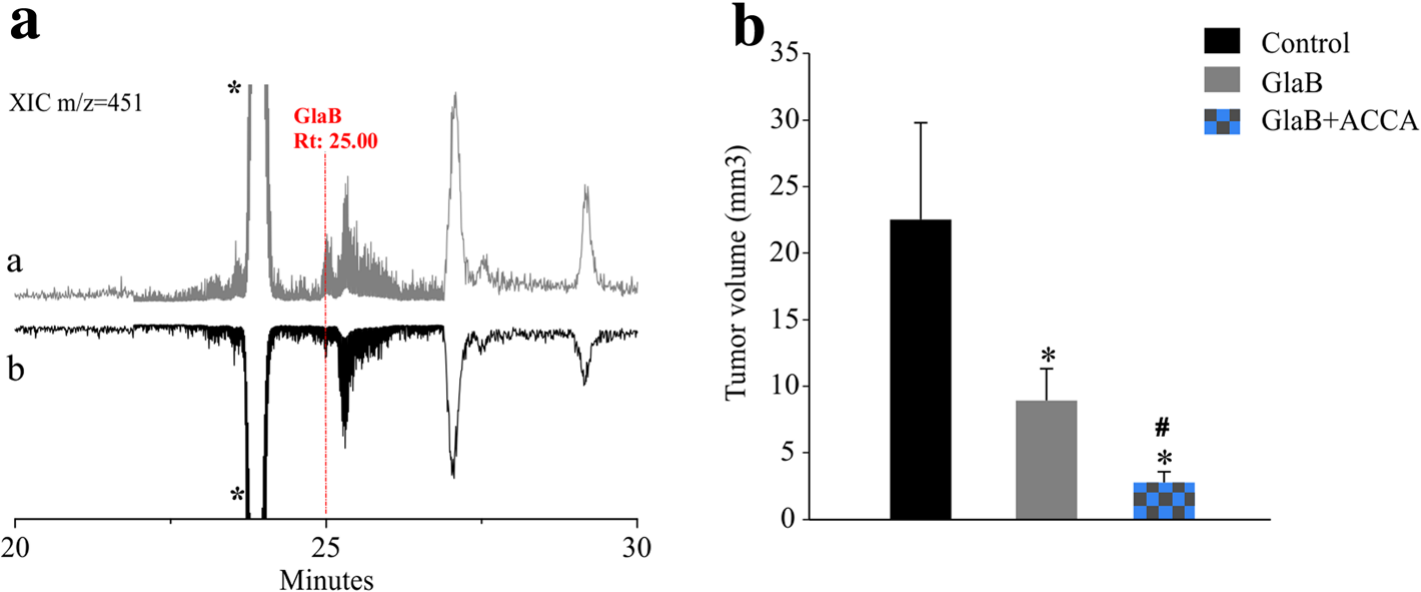
GlaB and GlaB/ACCA treatment in vivo is effective in glioma-bearing mice. **a** Extraction chromatograms (XIC) referred to brain extract of GlaB IN treated mice (a) and vehicle mice (b). GlaB peak at known retention time (Rt) of 25.00 min is shown and detected in IN treated sample and is missing in control. * The peak at Rt: 23.90 min is the isotopic abundance with m/z = 451 of an unknown peak with m/z = 449, present in all brain extracts*.* **b** Tumor volume (mm^3^) in GL261-bearing mice intra-nasally treated with GlaB (PEG-Cholane GlaB 1.44 mg/Kg), GlaB+ACCA (6 μl 33 mm) or vehicle (PEG-Cholane). Data are expressed as mean ± se, *p < 0.05 *vs* control, #*p* < 0.05 *vs* GlaB, One-way ANOVA *n* = 8

To verify brain delivery, we measured GlaB concentration in brain extracts of GlaB treated mice, by HPLC coupled with Electrospray Mass Spectrometry. The analytical method used the reversed phase chromathography and showed a high sensitivity (Additional file 1: Figure S2), leading to the quantification of GlaB present in brain extracts. Two hours after the first IN administration (GlaB/mPEG_5kDa_-Cholane formulation), we evaluated the presence of the drug (Fig. 4a, grey line) and quantified a mean value of 4.77 ± 0.35 μg/g of GlaB brain extract (Additional file 1: Figure S2 and S3). As shown in Fig. 4b, GlaB administration significantly reduced tumor volume, in comparison with vehicle-treated mice. GlaB/ACCA co-administration further reduced tumor volume compared with GlaB treatment alone, demonstrating that, in vivo, the blockade of lactate efflux potentiates the GlaB effect.

## Discussion

Since every cellular process has a defined metabolic fingerprint, the cell metabolomic approach is becoming one of the most-used tools to investigate crucial pathways in cancer development and drug-induced effects [24, 35–38]. The metabolism of cancer cells differs from that of normal cells: high-grade tumors, including GBM, must balance energy metabolism to synthesize the macromolecules essential for growth and progression relying on anaerobic metabolism, even under physiological oxygen levels [21, 39]. The tumor microenvironment also affects cancer cell proliferation, thus influencing treatment responses [40–42]. It has been reported that GBM cells in culture convert as much as 90% of glucose and 60% of glutamine they acquire into lactate or alanine [43]. Using an untargeted NMR approach, we measured the *endo*- and *exo*-metabolome of GlaB-treated and untreated cells and identified altered levels of metabolites over time.

While the changes of metabolite concentration levels at 12 h and 24 h can be explained as a direct and early consequence of GlaB effects on the metabolism, the changes at 48 h are of more difficult interpretation, being the combination of multiple effects. In particular, the intracellular increasing levels of lactate accompanied by the decreasing levels of glucose-6-phosphate demonstrate that GlaB exacerbates the Warburg effect. Consistently, GlaB-treated cells showed increased glucose uptake from the growing media and increased lactate excretion, likely to avoid cellular acidification. This metabolomic pattern, with the additional observation of both intracellular and extracellular increase in alanine concentration, reflect an enhanced glycolytic metabolism and high lactate production (Fig. 2). Pyruvate levels are also significantly increased by GlaB treatment at 12 h and 24 h. The higher intracellular levels of pyruvate can be explained as a sum of enhanced glycolysis and hindered TCA cycle. In fact, the alteration pattern of citrate, succinate, fumarate and malate levels suggests the presence of a truncated TCA cycle [43] in GlaB-treated cells. The absence of a fully functional TCA cycle in GlaB-treated cells is in accordance with the intracellular reduction of amino acids like glutamate, branched-chain amino acids and aspartate. The intracellular levels of glutamate progressively increased over time, but significantly reduced in GlaB-treated cells. This trend might suggest an inhibition of the mitochondrial enzyme glutaminase [44]. Moreover, the lower levels of isoleucine, leucine and valine in treated cells reinforce the hypothesis of reduced anaplerotic reactions as a consequence of a truncated TCA cycle [45–48]. The intracellular levels of aspartate and its derivative N-acetylaspartate are significantly decreased in GlaB-treated cells. Aspartate constitutes a key metabolic hub in the cells, being a major precursor for protein and nucleotide biosynthesis, as well as critical for the synthesis of other “non-essential” amino acids such as asparagine (below the NMR detection limit) and arginine (which also decreases upon GlaB treatment).

Metabolic data suggest that GlaB increased the glycolytic metabolism of GL261 cells, and the phosphorylation of proteins involved in the anabolic to catabolic transition, such as AMPK. These effects could be a consequence of the GlaB-induced reduction of intracellular levels of ATP [49], thus leading to the hypothesis that GlaB could favor energy stress in glioma. We also found that the increased lactate extrusion induced by GlaB treatment is important to prevent lactate “self-poisoning”, as already described for astrocytes and glioma cells [26]. Indeed, the blockade of lactate efflux by ACCA induced GL261 cell death by apoptosis, as already reported [25, 26]. The effect on cell viability was enhanced when lactate efflux was blocked simultaneously to GlaB treatment, with the induction of necrotic cell death. To test the translational potential of GlaB and GlaB/ACCA treatments, we used the syngeneic GL261 glioma model and delivered GlaB intranasally. Intranasal administration is commonly used to treat neurodegenerative diseases, to bypass the obstacle provided by the BBB [50]. With this treatment, GlaB accumulates in the brain, and efficiently reduces tumor volumes both in GlaB and in GlaB/ACCA treated mice.

## Conclusions

In this manuscript, we show for the first time the effect of an established Hh pathway inhibitor, GlaB, on glioma cell growth and metabolism, using in vitro and in vivo models of the pathology. In particular, we established by using NMR-based metabolomics analysis that GlaB treatment induces metabolic alterations in GL261 cells improving the glycolytic process over the TCA. Accordingly, we found that GlaB/ACCA treatment amplifies the anti-tumor effects of the single molecules, increasing cell necrosis and reducing tumor size in in vivo model of glioma. Our results could pave the road for future therapeutic strategies against human glioma and provide evidence that metabolomic analysis of cultured tumor cells is a valid method to study the metabolic alterations accountable for drug treatment. Although we found that the metabolites related to glycolysis are significantly associated with GlaB treatment of glioma cells, future works should be performed to further understand the molecular mechanism involved.

## Additional files

Additional file 1: **Figure S1.** The *endo*- and *exo*-metabolome assignment of GL261 cell line. Full region of the ^1^H-NMR spectra of cell lysates (**A**) and growing media (**B**) along with the assignment of the most intense metabolites, with the exclusion of water region. **Figure S2.** Chromatograms for estimated LOQ in UV and MS. (**A**) LOQ in UV determination is 1.48 ng of GlaB on column with a S/*N* = 16.7 for UV chromatogram and a S/*N* = 68.6 for MS trace. (**B**) LOQ in MS determination is 0.62 ng of GlaB on column with a S/*N* = 1.67 for UV chromatogram and a S/*N* = 13.6 for MS trace. **Figure S3.** Extract ion chromatograms (XIC) referred to IN treated sample (**A**) and spiked IN (0.45 μg/ml, 9 ng on column) treated sample (**B**). Both traces show the presence of GlaB peak (Rt: 25.00). * The peak at Rt: 23.90 min is the isotopic abundance with m/z = 451 of an unknown peak with m/z = 449, present in all brain extracts. **Figure S4.** Univariate analysis of the relative concentration levels of characteristic GL261 *endo*-metabolites. The box plots show the comparison of the relative intensities of the indicated metabolites between the untreated (CTR, light blue boxes) and treated (GlaB, orange boxes) groups at each time point. Metabolites whose concentration is significantly different (*p*-value < 0.05, by non-parametric Wilcoxon test) are marked with *. **Figure S5.** Univariate analysis of the relative concentration levels of GL261 *exo*-metabolites. The box plots show the comparison of the relative intensities of the indicated metabolites between the untreated (CTR, light blue boxes) and treated (GlaB, orange boxes) groups at each time point. Metabolites whose concentration is significantly different (*p*-value < 0.05, by non-parametric Wilcoxon test) are marked with *. The relative concentration levels of DMEM medium are also reported as Blank. **Figure S6.** Upfield region (0.75–1.8 ppm) of ^1^H-NMR CPMG spectra of cell lysates (*endo*-metabolome). Blue tracks, 12-CTR; red tracks, 24-CTR; purple tracks, 48-CTR; green tracks, 12-GLAB; orange tracks, 24-GLAB; cyan tracks, 48-GLAB. **Figure S7.** Representative FACS analyses showing the percentage of Annexin V/Propidium Iodide of cell population in GL261 untreated, treated with GlaB (5 μM), ACCA (250 μM) or both for 48 h. (DOCX 4706 kb)

Additional file 2: **Table S1.** List of the *endo*- and *exo*-metabolites assigned and analysed over time before and after GlaB treatment. Metabolites realised from GL261 cells after 48 h of treatment are reported separately. The Human Metabolome Database (HMDB) compound ID of each metabolite is also reported. **Table S2.** The HPLC gradient for the brain extracts analysis. All chromatographic runs were performed at a flow-rate of 0.4 ml/min. Solvent A was Water/Acetonitrile 90:10 with the 0.1% v/v of formic acid, and solvent B was Acetonitrile/Methanol 50:50 with the 0.1% v/v of formic acid. (DOCX 117 kb)

## Data Availability

The datasets used and/or analyzed during the current study are available on reasonable request.

## Abbreviations

ACCA: α-cyano-4-hydroxycinnamic acid
AMPK: AMP activated protein Kinase
BBB: Blood brain barrier
CNS: Central nervous system
CPMG: Carr-Purcell-Meiboom-Gill
Ct: Threshold cycle
Dhh: Desert hedgehog
DMEM: Dulbecco’s modified minimum essential medium
DMSO: Dimethyl sulfoxide
ESI: Electrospray ionization
FACS: Fluorescence-activated cell sorting
FBS: Fetal bovine serum
GBM: Glioblastoma
GL261: Murine glioma cells
GlaB: Glabrescione B
Hh: Hedgehog
HMDB: Human metabolome database
HPLC: High Performance Liquid Chromatography
Ihh: Indian hedgehog
IN: Intranasal
LOOCV: Leave-one-out cross-validation
LOQ: Limit of quantitation
MCTs: mono-carboxylate transporters
MS: Mass Spectrometry
MTT: 3-(4,5-Dimethylthiazol-2-yl)-2,5-diphenyltetrazolium bromide
NMR: Nuclear magnetic resonance
NOESY: Nuclear overhauser enhancement spectroscopy
PBS: Phosphate buffered saline
PCA: Principal component analysis
PI: Propidium iodide
Ptch: Patched
Shh: Sonic hedgehog
Smo: Smoothened
TCA: Tricarboxylic acid cycle
TMSP: Sodium 3-trimethylsilyl [2,2,3,3-^2^H_4_] propionate
