# Supplementary material for: 1H-NMR metabolomics reveals the Glabrescione B exacerbation of glycolytic metabolism beside the cell growth inhibitory effect in glioma
Source: Cell Commun Signal. 2019 Aug 28;17:108. doi: 10.1186/s12964-019-0421-8 (PMC6712882; doi:10.1186/s12964-019-0421-8)
Supplement: Supplementary file 1 — Figure S1. The endo- and exo-metabolome assignment of GL261 cell line. Full region of the 1H-NMR spectra of cell lysates (A) and growing media (B) along with the assignment of the most intense metabolites, with the exclusion of water region. Figure S2. Chromatograms for estimated LOQ in UV and MS. (A) LOQ in UV determination is 1.48 ng of GlaB on column with a S/N = 16.7 for UV chromatogram and a S/N = 68.6 for MS trace. (B) LOQ in MS determination is 0.62 ng of GlaB on column with a S/N = 1.67 for UV chromatogram and a S/N = 13.6 for MS trace. Figure S3. Extract ion chromatograms (XIC) referred to IN treated sample (A) and spiked IN (0.45 μg/ml, 9 ng on column) treated sample (B). Both traces show the presence of GlaB peak (Rt: 25.00). * The peak at Rt: 23.90 min is the isotopic abundance with m/z = 451 of an unknown peak with m/z = 449, present in all brain extracts. Figure S4. Univariate analysis of the relative concentration levels of characteristic GL261 endo-metabolites. The box plots show the comparison of the relative intensities of the indicated metabolites between the untreated (CTR, light blue boxes) and treated (GlaB, orange boxes) groups at each time point. Metabolites whose concentration is significantly different (p-value < 0.05, by non-parametric Wilcoxon test) are marked with *. Figure S5. Univariate analysis of the relative concentration levels of GL261 exo-metabolites. The box plots show the comparison of the relative intensities of the indicated metabolites between the untreated (CTR, light blue boxes) and treated (GlaB, orange boxes) groups at each time point. Metabolites whose concentration is significantly different (p-value < 0.05, by non-parametric Wilcoxon test) are marked with *. The relative concentration levels of DMEM medium are also reported as Blank. Figure S6. Upfield region (0.75–1.8 ppm) of 1H-NMR CPMG spectra of cell lysates (endo-metabolome). Blue tracks, 12-CTR; red tracks, 24-CTR; purple tracks, 48-CTR; green tracks, 12-GLAB; o [file 12964_2019_421_MOESM1_ESM.docx]

**Figure S1.** The *endo*- and *exo*-metabolome assignment of GL261 cell line. Full region of the ^1^H-NMR spectra of cell lysates (A) and growing media (B) along with the assignment of the most intense metabolites, with the exclusion of water region.


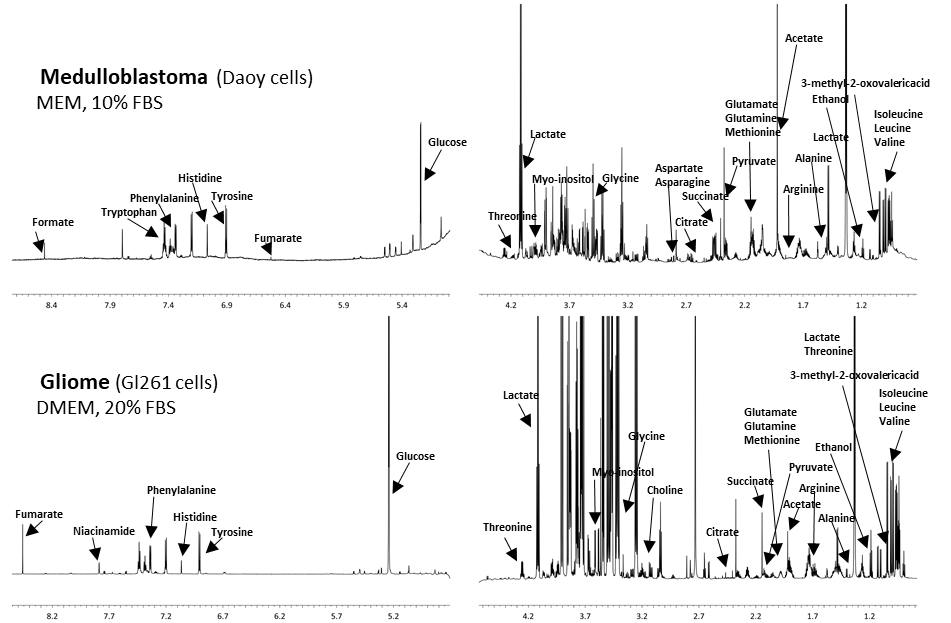

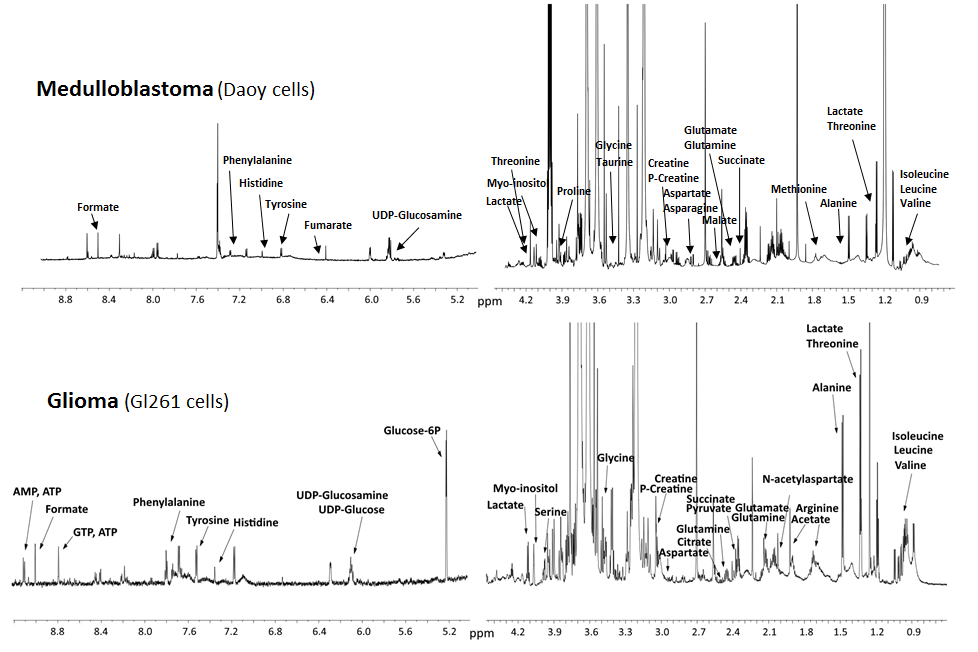


**A) *Endo-*metabolome**

**B) *Exo-*metabolome**

ppm

**A B**

LOQ UV: 1.48 ng on column LOQ MS: 0.62 ng on column


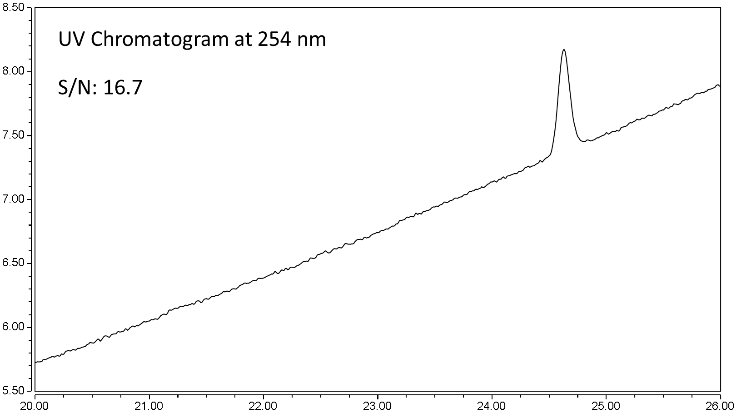

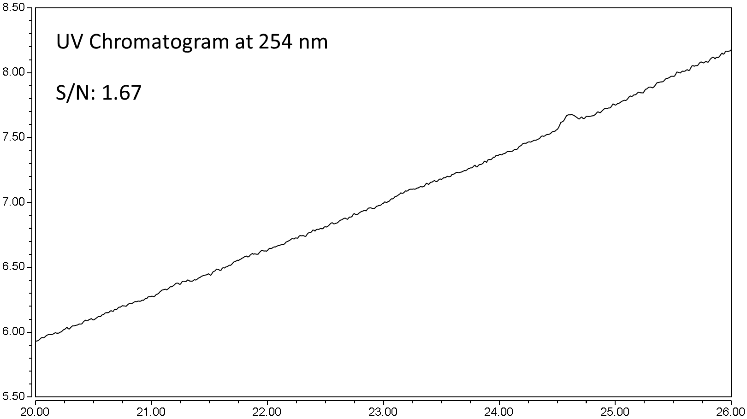


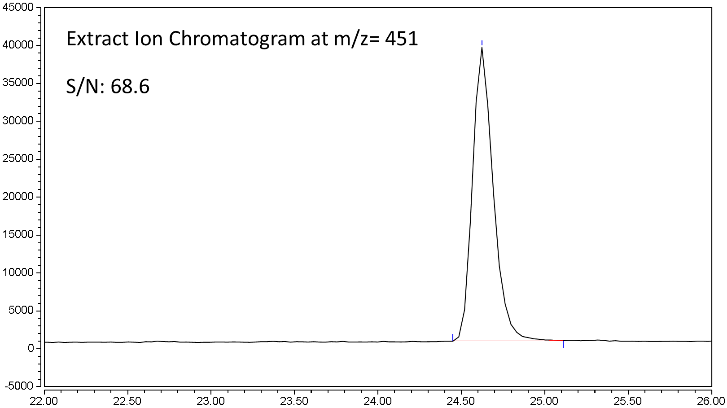

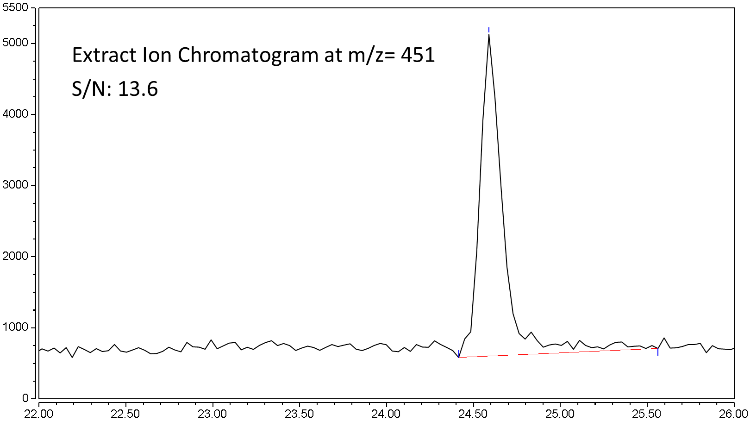


**Figure S2.** Chromatograms for estimated LOQ in UV and MS. (**A**) LOQ in UV determination is 1.48 ng of GlaB on column with a S/N= 16.7 for UV chromatogram and a S/N= 68.6 for MS trace. (**B**) LOQ in MS determination is 0.62 ng of GlaB on column with a S/N= 1.67 for UV chromatogram and a S/N= 13.6 for MS trace.

**
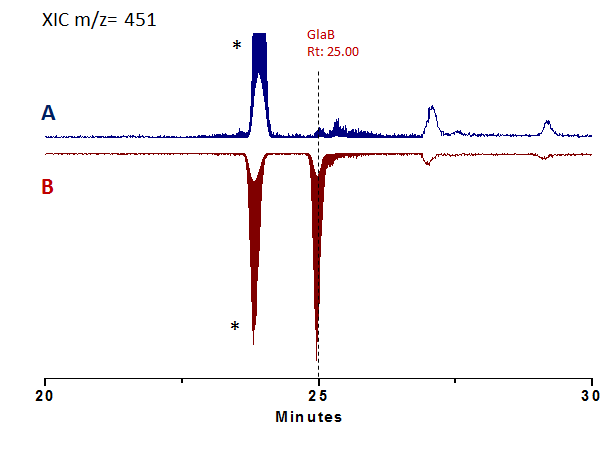
**

**Figure S3.** Extract ion chromatograms (XIC) referred to IN treated sample (**A**) and spiked IN (0.45 μg/ml, 9 ng on column) treated sample (**B**). Both traces show the presence of GlaB peak (Rt:25.00). * The peak at Rt: 23.90 min is the isotopic abundance with m/z=451 of an unknown peak with m/z=449, present in all brain extracts.


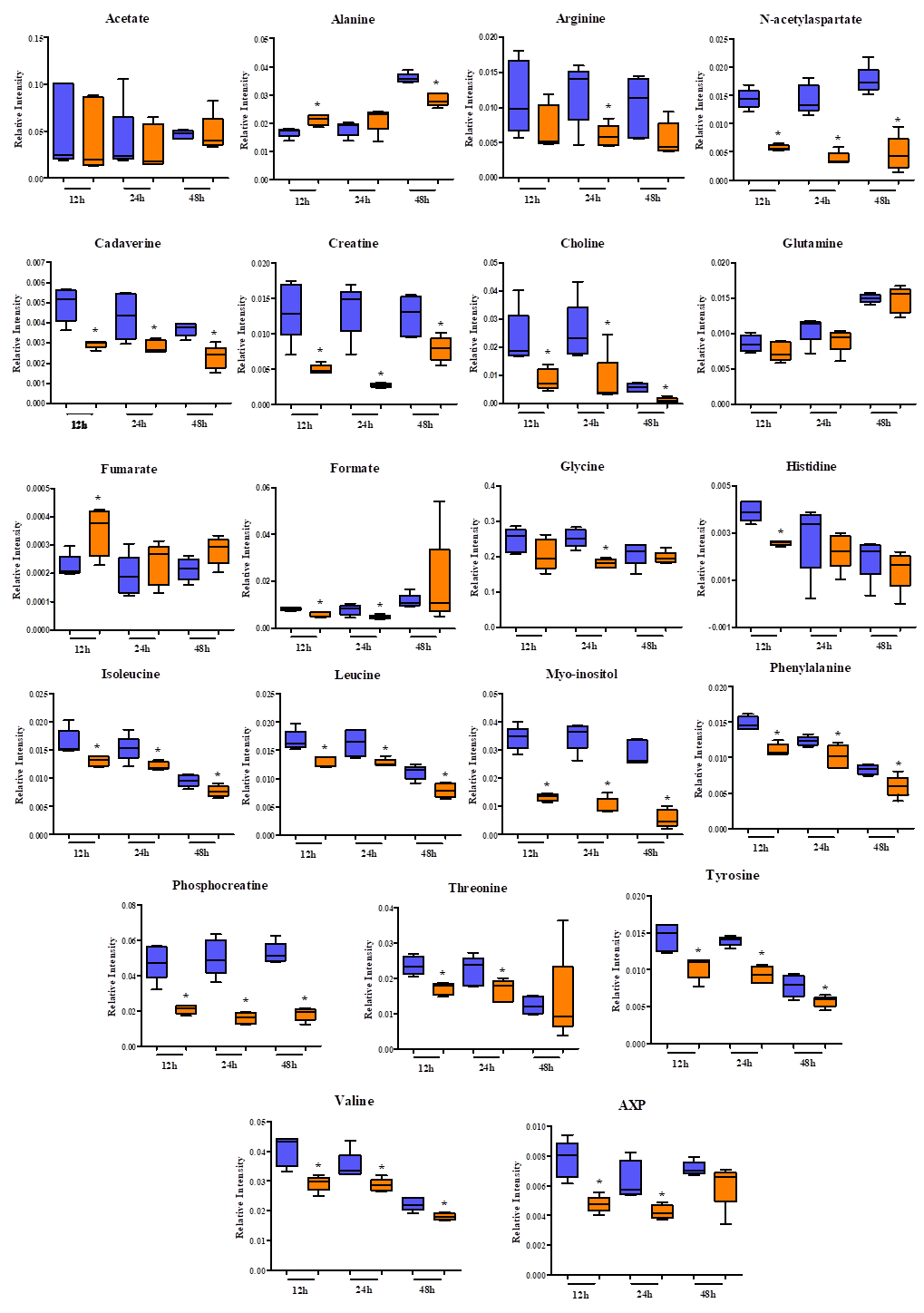


**Figure S4.** Univariate analysis of the relative concentration levels of characteristic GL261 *endo*-metabolites. The box plots show the comparison of the relative intensities of the indicated metabolites between the untreated (CTR, light blue boxes) and treated (GlaB, orange boxes) groups at each time point. Metabolites whose concentration is significantly different (*p*-value < 0.05, by non-parametric Wilcoxon test) are marked with *.

SK1 expression in ovarian cancer cells affects

the metabolomic proﬁles


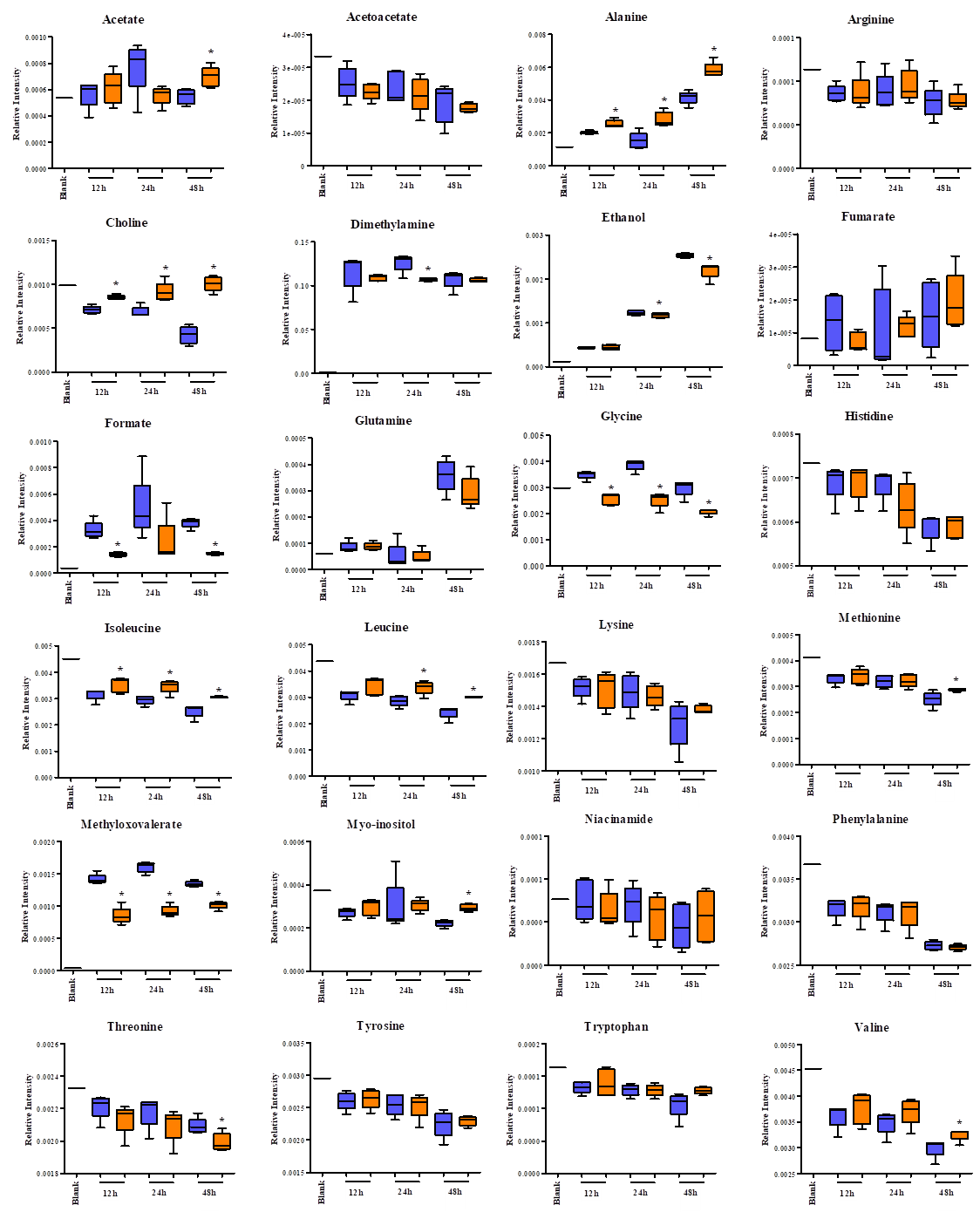


**Figure S5.** Univariate analysis of the relative concentration levels of GL261 *exo*-metabolites. The box plots show the comparison of the relative intensities of the indicated metabolites between the untreated (CTR, light blue boxes) and treated (GlaB, orange boxes) groups at each time point. Metabolites whose concentration is significantly different (*p*-value < 0.05, by non-parametric Wilcoxon test) are marked with *. The relative concentration levels of DMEM medium are also reported as Blank.


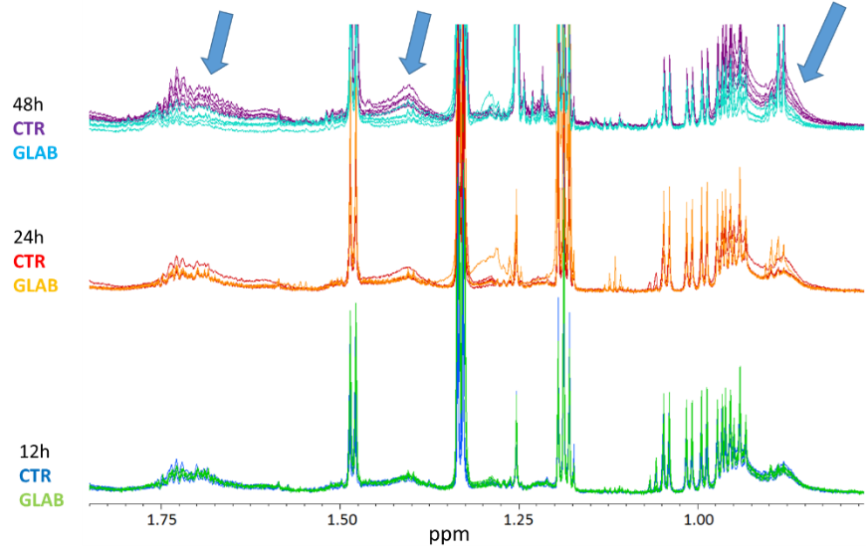


**Figure S6.** Upfield region (0.75 – 1.8 ppm) of ^1^H-NMR CPMG spectra of cell lysates (*endo*-metabolome). Blue tracks, 12-CTR; red tracks, 24-CTR; purple tracks, 48-CTR; green tracks, 12-GLAB; orange tracks, 24-GLAB; cyan tracks, 48-GLAB.

*
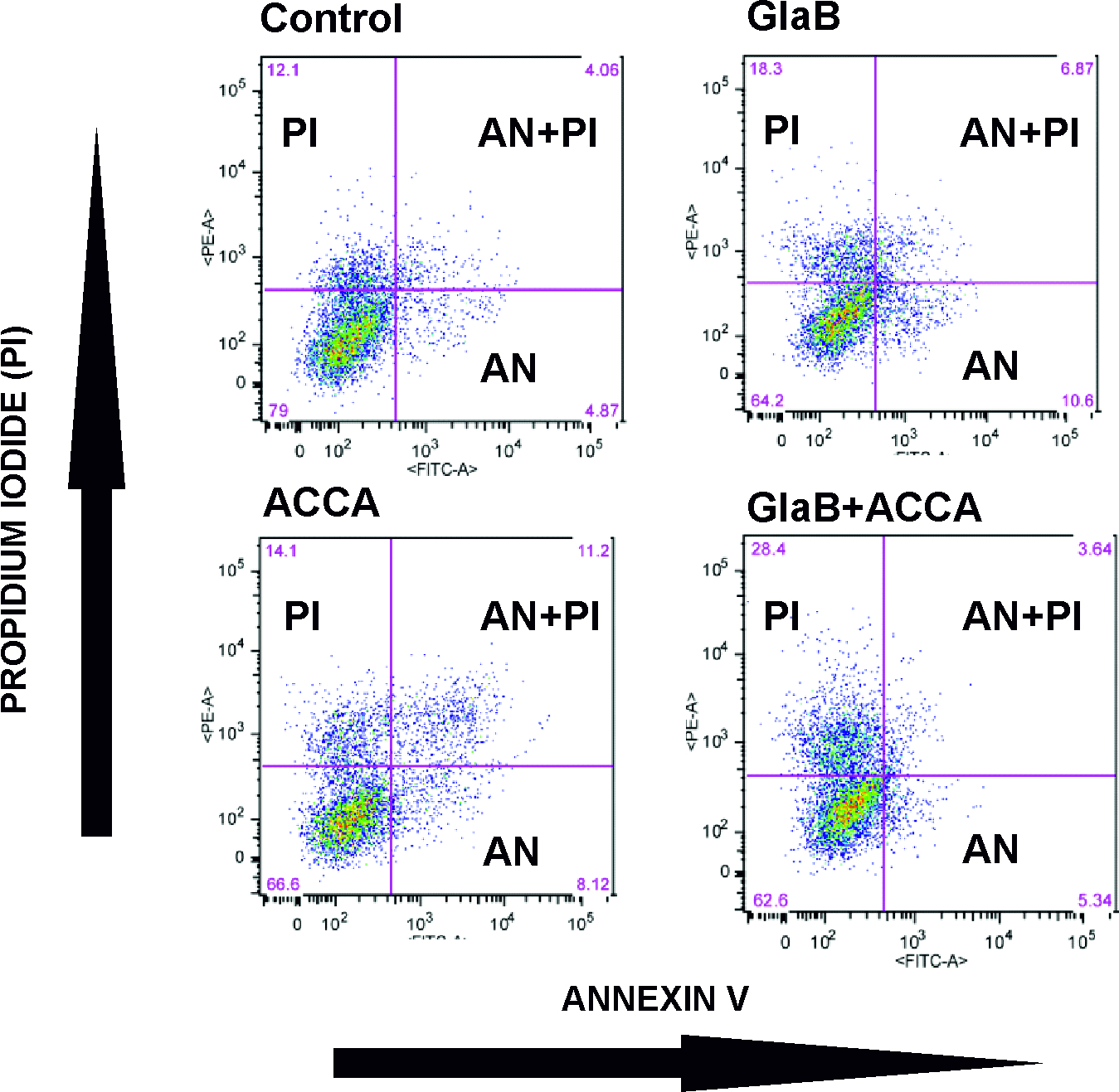
*

**Figure S7.** Representative FACS analyses showing the percentage of Annexin V/Propidium Iodide of cell population in GL261 untreated, treated with GlaB (5 µm), ACCA (250 µm) or both for 48h.
