# Supplementary material for: 1H-NMR metabolomics reveals the Glabrescione B exacerbation of glycolytic metabolism beside the cell growth inhibitory effect in glioma
Source: Cell Commun Signal. 2019 Aug 28;17:108. doi: 10.1186/s12964-019-0421-8 (PMC6712882; doi:10.1186/s12964-019-0421-8)
Supplement: Supplementary file 2 — Table S1. List of the endo- and exo-metabolites assigned and analysed over time before and after GlaB treatment. Metabolites realised from GL261 cells after 48 h of treatment are reported separately. The Human Metabolome Database (HMDB) compound ID of each metabolite is also reported. Table S2. The HPLC gradient for the brain extracts analysis. All chromatographic runs were performed at a flow-rate of 0.4 ml/min. Solvent A was Water/Acetonitrile 90:10 with the 0.1% v/v of formic acid, and solvent B was Acetonitrile/Methanol 50:50 with the 0.1% v/v of formic acid. (DOCX 117 kb) [file 12964_2019_421_MOESM2_ESM.docx]

**
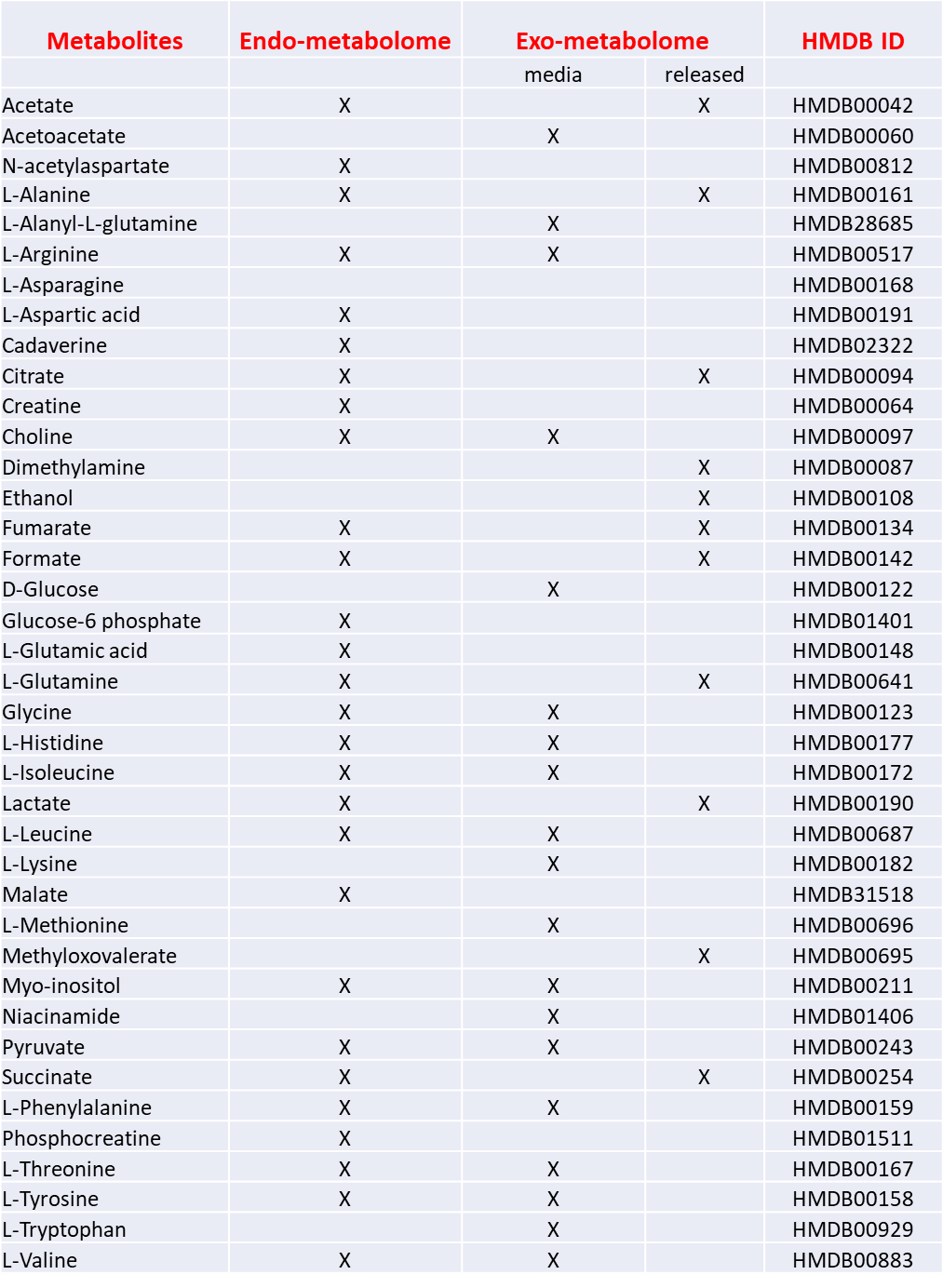
**

**Table S1.** List of the *endo*- and *exo*-metabolites assigned and analysed over time before and after GlaB treatment. Metabolites realised from GL261 cells after 48h of treatment are reported separately. The Human Metabolome Database (HMDB) compound ID of each metabolite is also reported.

| TIME (min) | FLOW (ml/min) | % Solvent B |
| --- | --- | --- |
| 0 | 0.4 | 20 |
| 3 | 0.4 | 20 |
| 4 | 0.4 | 50 |
| 37 | 0.4 | 100 |
| 42 | 0.4 | 100 |
| 44 | 0.4 | 20 |
| 48 | 0.4 | 20 |

**Table S2.** The HPLC gradient for the brain extracts analysis. All chromatographic runs were performed at a flow-rate of 0.4 ml/min. Solvent A was Water/Acetonitrile 90:10 with the 0.1% v/v of formic acid, and solvent B was Acetonitrile/Methanol 50:50 with the 0.1% v/v of formic acid.
